# Monolayer Graphene Transfer onto Hydrophilic Substrates: A New Protocol Using Electrostatic Charging

**DOI:** 10.3390/membranes10110358

**Published:** 2020-11-20

**Authors:** Feras Kafiah, Tahar Laoui, Emad Abdelsalam, Muataz Ali Atieh, Zafarullah Khan, Malek Alkasrawi

**Affiliations:** 1 School of Engineering Technology, Al Hussein Technical University, Amman 11831, Jordan; feras.kafiah@htu.edu.jo (F.K.); emad.abdelsalam@htu.edu.jo (E.A.); 2 Department of Mechanical and Nuclear Engineering, University of Sharjah, Sharjah 27272, UAE; mhussien@sharjah.ac.ae; 3 Department of Mechanical Engineering, King Fahd University of Petroleum & Minerals, Dhahran 31261, Saudi Arabia; zukhan@kfupm.edu.sa; 4 Department of Engineering, Wisconsin Institute for Sustainable Technology (WIST), College of Natural Resources, University of Wisconsin-Stevens Point, Stevens Point, WI 54481, USA

**Keywords:** graphene transfer, graphene membranes, microfiltration membranes, hydrophilic surfaces, electrostatic charging

## Abstract

In the present work, we developed a novel method for transferring monolayer graphene onto four different commercial hydrophilic micro/ultra-filtration substrates. The developed method used electrostatic charging to maintain the contact between the graphene and the target substrate intact during the etching step through the wet transfer process. Several measurement/analysis techniques were used in order to evaluate the properties of the surfaces and to assess the quality of the transferred graphene. The techniques included water contact angle (CA), atomic force microscopy (AFM), and field emission scanning electron microscopy (FESEM). Potassium chloride (KCl) ions were used for the transport study through the developed graphene-based membranes. The results revealed that 70% rejection of KCI ions was recorded for the graphene/polyvinylidene difluoride (PVDF1) membrane, followed by 67% rejection for the graphene/polyethersulfone (PES) membrane, and 65% rejection for graphene/PVDF3 membrane. It was revealed that the smoothest substrate was the most effective in rejecting the ions. Although defects such as tears and cracks within the graphene layer were still evolving in this new transfer method, however, the use of Nylon 6,6 interfacial polymerization allowed sealing the tears and cracks within the graphene monolayer. This enhanced the KCl ions rejection of up to 85% through the defect-sealed graphene/polymer composite membranes.

## 1. Introduction

The unique structure of graphene and its distinct physical and chemical properties offer a great prospect for numerous industrial applications such as touch screens, solar cells, smartphones, nanomaterials, sensors, and batteries [1,2,3]. In membranes based applications, various approaches are being explored by researchers that target the permeability and fouling issues. The main goal of the different approaches is to find alternative membrane material which is inherently free from the limiting factors which hinder the ultrafast water transport through the membranes [4,5,6,7]. Monolayer graphene, however, is almost totally impermeable even to helium atoms [8,9], and the key factor for its use as a separation membrane, especially for water purification applications, is to transfer and support the CVD-grown monolayer graphene to the targeted substrates [10,11,12]. Graphene utilization in a wide range of applications is dependent upon the quality of graphene transfer from the growth substrate to the application substrate. One of the main challenges with graphene, is not only to produce a high-quality monolayer, but also to yield a defect free transferred graphene. The presence of defects [13], impurities [14], multiple domains [15], structural disorders [16], wrinkles [17] in the graphene layer imposes adverse effect on its properties and performance. In electronic applications, the major bottleneck is the need for large size graphene layer/sheet, which is only possible when graphene is fabricated using Chemical Vapor Deposition (CVD) process.

Although there are many graphene preparation methods, there is no single method that is adequate for all applications. That is because each method comes with advantages and challenges [18,19]. For example, the CVD process is typically used to grow graphene over copper (Cu) substrate. [20]. The CVD process uses heat in order to decompose the carbon atoms from a supply gas that is enriched with carbon, then promotes the growth of graphene over catalyst substrate [21]. Having said that, the CVD process is favorbale because it is not considered costly and it is capable of producing a high-quality graphene, with large growth area [22,23,24]. Additionally, the CVD process produces graphene/Cu sheets that can be directly used in applications or have graphene transferred to other substrates [25,26]. Furthermore, the CVD method allows synthesis of graphene onto different transition metals: iridium [27,28], ruthenium [27,29,30,31], nickel [32,33,34,35,36], palladium [37,38,39], cobalt [38], rhenium [40], platinum [41], and copper [42]. Graphene growth with large surface area is particularly obtained with copper or nickel substrates [30]. On the other hand, many of the application substrates are not metals, rather polymeric materials, hence, it is necessary to transfer the high-quality grown graphene from the metal substrate to the targeted polymeric substrate without degrading its quality. Polymeric substrates could be classified according to their surface water wettability into hydrophilic and hydrophobic substrates. Substrate selection primarily depends on the application. Since graphene is hydrophobic, it adheres better to the hydrophobic surfaces compared to the hydrophilic counterpart. Therefore, it is a challenge to transfer graphene onto hydrophilic substrates [43,44,45]. Belayeva et al. [46] discussed and presented the lowest water contact angle values measured on graphene. They suggested that the wettability of graphene was governed by the wettability of the underlying catalyst support. Contact angle values were reported ranging from 33° for graphene on silicon to greater than 90° for graphene on Cu and other substrates [47].

Graphene transfer could be classified into two main categories: the wet transfer method (WTMs) -which is commonly used- and the dry transfer method (DTM). The WTM uses etching solution in order to remove the Cu substrate. Typical etching solutions used are: iron nitrate [26], ammonium persulfate (APS) [48], and ferric chloride [33]. During the transfer process, it is likely that graphene tends to tear and crack. However, to reduce this issue, a good adhesion must exist between the graphene layer and the target substrate. Furthermore, the strength of the adhesion is very dependent on substrate’s hydrophobicity and surface roughness. In the case of hydrophilic surfaces, the etchant solution penetrates easily between the polymeric substrate and the graphene/Cu leading to the detachment of the two surfaces.

In this work, we introduced a new efficient protocol to transfer graphene monolayer onto polymeric hydrophilic substrates, this being a vital step towards developing a high-performance membrane for water purification applications. The method utilized electrostatic charging to maintain the contact of graphene/polymeric substrates intact during the etching step. The quality of transferred graphene was characterized using field emission scanning electron microscopy (FESEM), and the performance of graphene/polymeric substrate composites was evaluated using KCl ionic transport study. 

## 2. Materials and Procedures 

### 2.1. Materials

Four different hydrophilic commercial substrates were utilized in this study, Table 1. One polyethersulfone (PES) and three polyvinylidene difluoride (PVDF 1, PVDF 2 and PVDF 3) were purchased from Novamem Advance Separations Company, Switzerland. Althought the procured polyvinylidene difluoride substrates have the same pore size of 10 nm as shown in Table 1, the substrates vary in their pore structure and their surface weteability attributes. To prepare the Cu etchant, ammonium persulfate (APS) with 25% (*wt/vol*) was mixed with de-ionized water. The APS was purchased from Eurostar Scientific LTD, in Germany. The copper etchant was utilized to remove Cu during the transfer process. The transport studies were performed using potassium chloride salt (KCl), which was procured from Merck Chemicals (Eschborn, Germany).

ACS Material, a USA based company, supplied the CVD monolayer graphene. To ensure that the monolayer graphene covered the Cu substrate, Raman spectroscopy analysis was used. 

### 2.2. Monolayer Graphene Transfer using Electrostatic Charging

**Figure 1:**
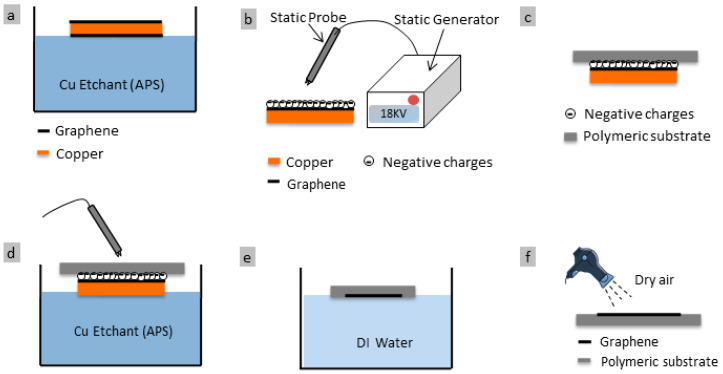
Diagram showing the process of transferring graphene onto hydrophilic substrate; (**a**) one-sided graphene is removed after placing it for seven minutes in APS etchant, (**b**) the process of charging Cu/graphene with negative ions using 18 kV static generator gun, (**c**) attaching Cu/graphene to the polymeric substrate (**d**) Cu surface etching; this step is accomplished allowing the substrate to float (for 20 min) in a 25% APS Cu etchant, (**e**) graphene/polymeric substrate washing, for 10 min using de-ionized water, (**f**) air drying of graphene/polymeric substrate composite.

The Cu etchant has a quick tendency to wet hydrophilic substrates. Hence, the wet etching transfer methods are not suitable. To address this issue, we developed a new approcah to allow the graphene to be transferred onto hyrdophilic substrates. The new approcah used a 5% (*wt/vol*) diluted APS etchant in order to remove the one-sided graphene. The graphene was floated over the etchant during this process, as shown in Figure 1a. The next step in the process was to attach the Cu/graphene to the polymeric substrate by exposing the composite stacks to 18 kV negative charges using an electrostatic generator and with a special electrode placed at a distance of 5 cm away from the substrate (Figure 1b,c). The electrostatic generator (model: SIMCO 18kV) was procured from SIMCO Ion Company (Hatfield, PA, USA). Next, a 25% (*wt/vol*) concentrated APS etchant was used to remove Cu from the composite, by floating the Cu/graphene/substrate over the etchant. The graphene stayed attached to the substrate during this step, as displayed in Figure 1d. Etching took about 20 min to complete.

It is crucial to keep the electrostatic charging ON over the composite layers. This is crucial in order to keep the graphene and polymeric substrate in contact during the entire transfer process. Failure to do this, the etchent may seep in and cause detachment. The surface of graphene/Cu foil is attracted to the polymeric substrate by an electrostatic force. The negative charges accumulated at the graphene surface are attracted to the positive charges at the polymeric substrate surface. The resultant electrostatic potential is proportional to the amount of accumulated charges between the contacted surfaces. By keeping the electrostatic charging ON during the Cu etching step, this prevents the charges dissipation that could take place if the electrostatic generator is turned off. The final step in this process is to wash the graphene/substrate by de-ionized water bath for 10 min and then the let it air dry, as shown in Figure 1e,f. 

### 2.3. Ionic Transport Study

**Figure 2:**
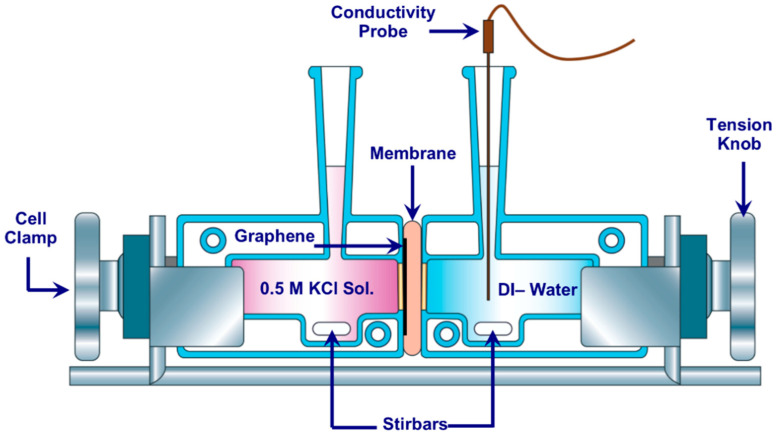
Illustration of the Side-bi-Side diffusion cell.

In order to assess the quality of the transferred monolayer graphene, KCl ions transport study was performed through the developed composite membranes. For this purpose, we utilized a dedicated diffusion cell (Side-bi-Side) purchased from Permegear Inc., Pennsylvania, USA as shown in Figure 2. The left side of the cell was placed with a 0.5 M KCl solution, while de-ionized water was placed in the right of the cell. The cell combines two glass chambers (7 mL each) setting on a stand using tension knob. Each side of the cell has a 3 mm parallel orifice, the graphene layer is facing the left orifice when both chambers are clamped and sealed together. 

Before placing the graphene memberane in the cell, both chambers of the cell were cleaned using de-ionized water, and then allowed to air-dry. The active side of the graphene was placed, so that it was facing the the left side chamber. In order to ensure that no water bubles accumelate at the surface of the memberane, both cell’s chambers were washed with ethanol, after tighening the cell. Furthermore to ensure that no excess ethanol was trapped on the membrane, a 0.5 M KCl solution was used to wash the left side chamber that faces the membrane, and using de-ionized water to wash the membrane side facing the right chamber. This was repeated for three times. 

Using the osmosis process caused by the concentrtion difference, the KCl ions moved from the left chamber to the right chamber diffusing through the composite membrane layers. In order to reduce the effect of concentration polarization during the diffusion, magnetic stirring was employed on the solutions in both side of the cell. In order to aquire and monitor the diffusion rate of the ions, we measured the change in conductivity with time using a special condctivity probe. The elctrode probe was manufactured by eDAQ Pty Ltd (Denistone East, New South Wales, Australia). The electrode was placed in the de-ionized water (right chamber) as shown in Figure 2. The conductivity was recorded every 30 s for 10 min. The acquired measurements were plotted as a time vs. conductivity graphs. The slope of the graph was then compared with that of a bare substrate (no graphene). In order to calculate the percentage of the ions’ rejection, we determined the difference between the bare substrate slope and the composite slope, followed by dividing the results by the slope of the bare substrate. Graphene is impermeable even for helium [8,9]. Having said that, the higher ions rejection, the better the quality of prepared graphene. Once graphene was transferred with a high quality and being able to reject any speices, Sean et al. [12] proposed to use ion bombardment process to open up the targeted pore size required by the application field.

### 2.4. Interfacial Polymerization Process

**Figure 3:**
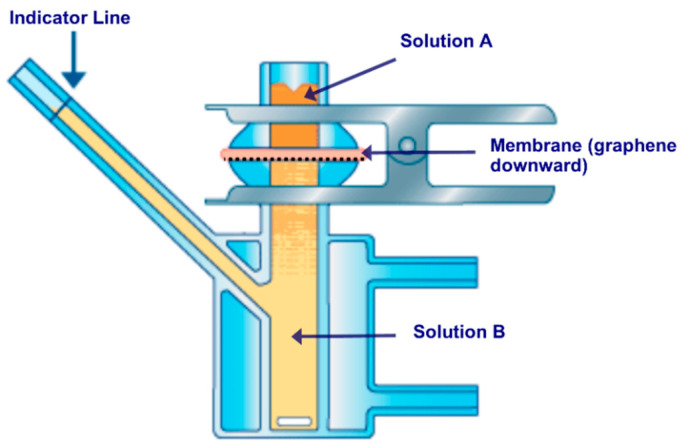
Schematic of Franz cell utilized for interfacial polymerization process.

Defects such as cracks and tears might arise inside graphene layer throughout the transfer process. These defects need to be repaired- through plugging and sealing- to produce a defect-free graphene membrane. Defects plugging was performed using interfacial polymerization (IP) of Nylon 6.6 in defects spaces according to the procedure described in [49]. In order to perform the interfacial polymerization process, a Franz cell was used. The cell was manufactured by Permegear Inc. in the USA. Illustration of the process is shown in Figure 3, and the cell consists of two stacked up chambers. The top chamber was filled with a 27 mM Adipoyl chloride (APC) solution mixed with hexane, referred to as solution A. The bottom chamber was filled with 45 mM Hexamethylenediamine (HMDA) solution mixed with de-ionized water, referred to as solution B. Solution B was labeled with Texas red (TR) fluorescent dye, which, after the polymerization process, clearly delineated the presence of Nylon 6.6 zones on the membrane surface. Nylon 6.6 polymerization took place at the interface when Solution A from the upper chamber traveled downwards and penetrated through the graphene defects to meet Solution B at the other side of the graphene monolayer. Since graphene was impermeable, the only way for the two monomer solutions to meet each other was through the defect sites in graphene (tears and cracks). The bottom chamber was filled with solution B, and the graphene composite was laid over the cell’s orifice. The upper chamber was then placed over the other side of composite membrane and filled with solution A. The two chambers were then tightly held together using a special stainless steel clamp.

The IP process was performed only for 5 min. Once completed, the upper part was rinsed with hexane and ethanol multiple times. This is to ensure the removal of any residual species within the composite membrane. Sealed graphene composite membrane was then removed and washed with de-ionized and ethanol water baths before air drying.

## 3. Results and Discussion

The surface characteristics of the considered substrate affect the quality of transferred graphene, which in turn impacts the performance of the developed membrane. A smooth surface with a minimal average root mean square (rms) is preferred for the graphene/polymer membrane over a rough surface. The value of the contact angle is another measuring criterion related to the final product quality. The degree of hydrophobicity is crucial to maintain the graphene/substrate contact during the transfer process. Stable and reliable graphene/substrate contact prevents etchant solution from penetrating and delaminating the adhered layers. In wet graphene transfer methods, it is rather difficult for graphene to be attached onto a hydrophilic surface because it can be easily wetted by the etchant solution. This wetting action causes delamination even before the completion of the transfer process. 

**Figure 4:**
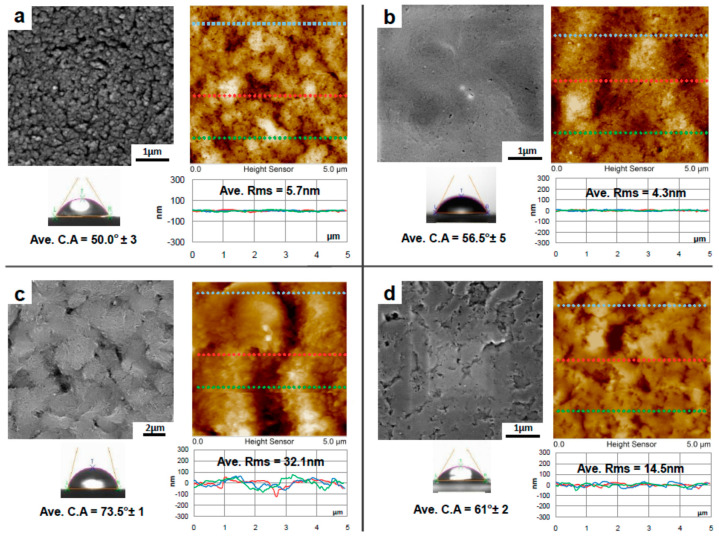
Surface characteristics of the as-received polymeric substrates: FESEM micrographs with 5 × 5 µm^2^, AFM images and three section profiles taken from each substrate, average RMS values, and average contact angle (CA) for (**a**) PES, (**b**) PVDF 1, (**c**) PVDF 2 and (**d**) PVDF 3.

Four commercial hydrophilic substrates were utilized in the new transfer process. FESEM and AFM micrographs analyses were carried out in order to characterize the as-received substrates, in terms of their surface morphology, and surface roughness (root mean square) as shown in Figure 4. The contact angle (CA) was measured to assess the degree of hydrophilicity of the substrates.

As shown in Figure 4, 10 nm pore size PVDF 1 substrate has the smoothest surface (RMS = 4.3 nm) with average contact angle ~ 56.5^°^. PVDF 2 has the roughest surface (RMS = 32.1 nm) with average contact angle ~73.5°.

Table 2 summarizes the surface characteristics of all substrates. The CA decreases when the surface is smoother, smooth surfaces are more hydrophilic. This is consistent with the findings reported in [50,51], confirming that smoother surfaces exhibit higher hydrophilicity as long as the surface contains hydrophilic functional groups such as methyle. Based on the contact angle measurements, all substrates are considered hydrophilic since their CAs are less than 90°. However, the variation of the CA values is related to the amount of electronegative functional groups at the polymer surface that establishes hydrogen bonding with water.

### 3.1. Graphene Transfer onto the Hydrophilic Substrates

**Figure 5:**
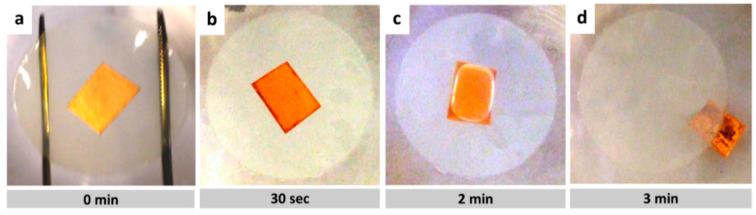
Graphene/Copper detachment in wet transfer process and without electrostatic charging, (**a**) substrate/graphene/Cu composite is placed, floated, over the APS etching solution, (**b**) about 30 s later, the etchant solution begins to pentrate, from edges (dark regions), (**c**) large air bubble between Cu/graphene and substrate (**d**) Cu/graphene and substrate detachment is completed.

When a substrate surface is hydrophilic, the etchant can penetrate between the Cu/graphene layer and the polymeric substrate, causing graphene to detach from the composite. The process of graphene detachment is shown in Figure 5. First, the substrate/graphene/Cu composite is placed, floated, in the APS etching solution as shown in Figure 5a. About 30 s later, the etchant solution begins to pentrate between the substrate and graphene/Cu, as shown in Figure 5b. After almost two minutes, an air bubble is then formed between graphene/Cu and the polymeric substrate as shown in Figure 5c. The bubble expands and pushes graphene/Cu away from the polymeric substrate leading to complete detachment, as shown in Figure 5d.

**Figure 6:**
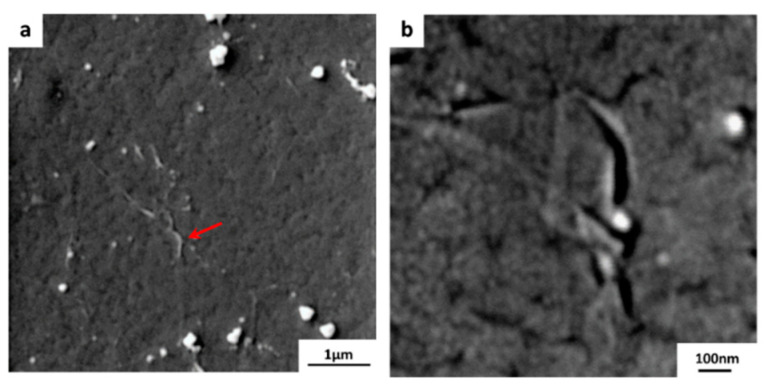
FESEM images of the graphene/PES composite membrane. The monloayer graphene is about ~ 1 × 1 cm^2^, (**a**) a wrinkle within the graphene layer is indicated by the red arrow, (**b**) higher magnification image.

The results of the above process confirm that having sufficient hydrophobicity for the polymeric substrate is necessary to ensure that etchant solution does not penetrate between the graphene and the substrate interface. To avoid the delamination process coined with the conventional transfer process, the method explained in Section 2.2 was used in order to transfer graphene onto the hydrophilic substrates as shown in Figure 1. It is very critical to keep electrostatic charging during the whole transfer process in order to prevent etchant from penetrating and delaminating the composite layers. By using the electrostatic charging, monolayer grpahene was successfully transferred to all four hydrophilic substrates. FESEM images for graphene/PES composite are shown in Figure 6. The size of the monloayer graphene is about 1 × 1 cm^2^. A high quality graphene can be easily be seen in the Figure 6a, where graphene wrinkle is marked with a red arrow. Copper residues are also noticed. A higher magnification micrograph is included (Figure 6b) in order to show the high quality graphene along with the sufrace morpology of PES substrate.

**Figure 7:**
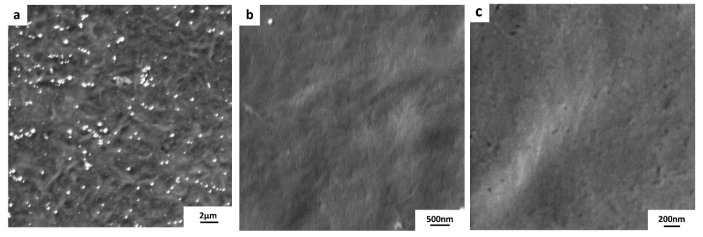
FESEM micrographs of the transferred graphene onto PVDF 1 substrate. The size of the transferred graphen is ~ 1 × 1 cm^2^. (**a**) white spots are clearly visible as indication of copper residues. (**b**,**c**) show a higher magnification images for the graphene/PVDF 1 composite surface.

Figure 7 displays the graphene/PVDF 1 composite. PVDF 1 substrate has the smoothest surface with RMS = 4.6 ± 0.7. FESEM micrographs indicate a high quality of transferred graphene with a good coverage over the PVDF substrate area (~ 1 × 1 cm^2^). Although copper residues (appearing as white spots in Figure 7a) are dispersed over the surface, they did not affect the quality of the transferred graphene monolayer, as shown in Figure 7b,c.

**Figure 8:**
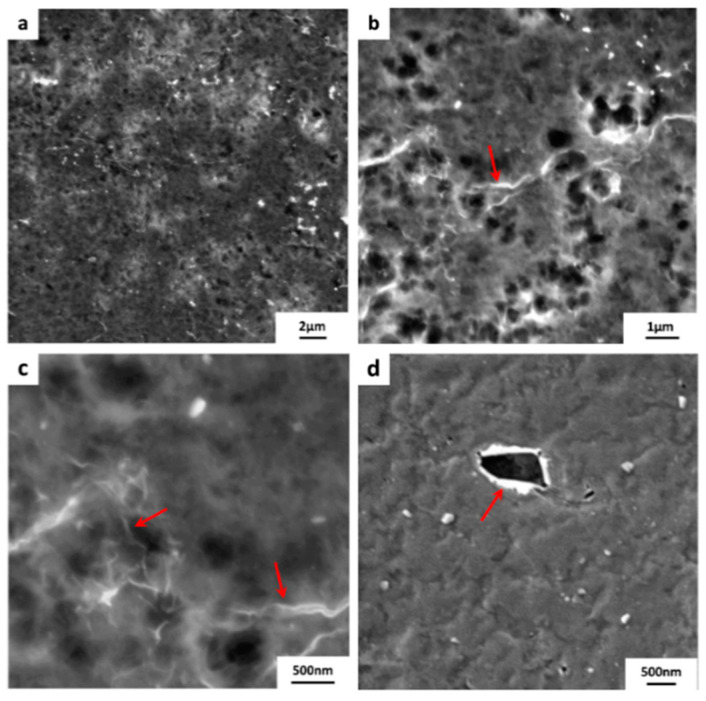
FESEM micrographs of the transferred graphene onto PVDF 2 substrate. The size of the transferred graphen is ~ 1 × 1 cm^2^. Tears and wrinkles within the graphene monolayer are labeled with red arrows. (**a**–**d**) are micrographs of graphene/PVDF 2 surface taken at different locations and magnifications.

The substrate PVDF 2 has the roughest surface when compared with PVDF 1 and PES. It is expected that the substrate roughness will induce tears and cracks on the monolayer graphene, however, the quality of transferred graphene is still acceptable. Figure 8a–d shows the FESEM images of the graphene transferred. Red arrows indicate wrinkles and tears distributed in the graphene layer.

**Figure 9:**
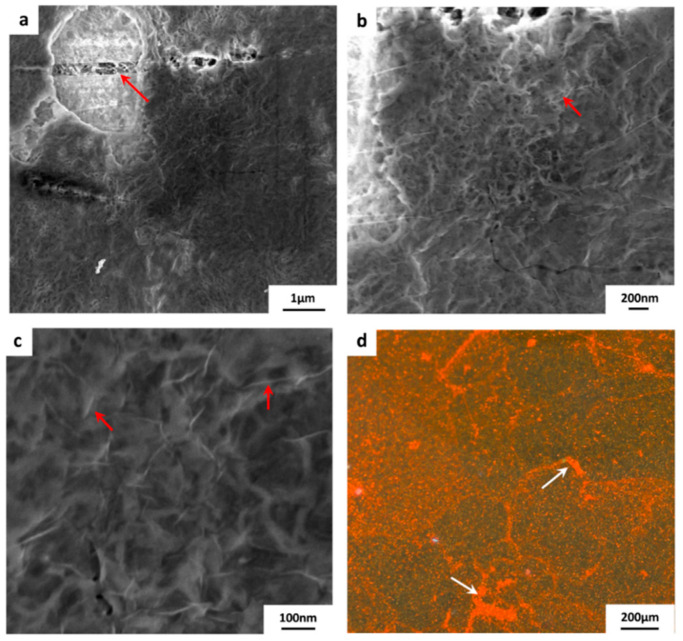
FESEM images of the graphene/ PVDF 3 composite membrane, red arrows indicate: (**a**) tears in the graphene layer, (**b**,**c**) wrinkles within graphene layer. (**d**) A magnified fluorescent micrograph showing Nylon 6.6 zones developed by IP process, white arrows indicate sealing of tears and cracks in graphene layer with Nylon 6.6.

Figure 9 shows the images of graphene/PVDF 3 composite. This substrate has surface roughness and hydrophilicity values in between PVDF 1 and 2. Tears and cracks defects are found in the graphene monolayer, Figure 9a. Wrinkles within graphene are seen in a higher magnification images indicated by red arrows in Figure 9c. This provides evidence on the presence of graphene on top of polymeric substrates. 

As mentioned in Section 2.4, solution B in the IP process was labeled with Texas red (TR) fluorescent dye, which, after the polymerization process, clearly delineated the presence of Nylon 6.6 zones on the membrane surface. A magnified fluorescent micrograph (Figure 9d) shows the graphene domains that contained tears and cracks due to the transfer process and were then sealed with Nylon 6.6 by the IP process.

### 3.2. Ionic Transport through Graphene/Substrate Membrane Composites 

**Figure 10:**
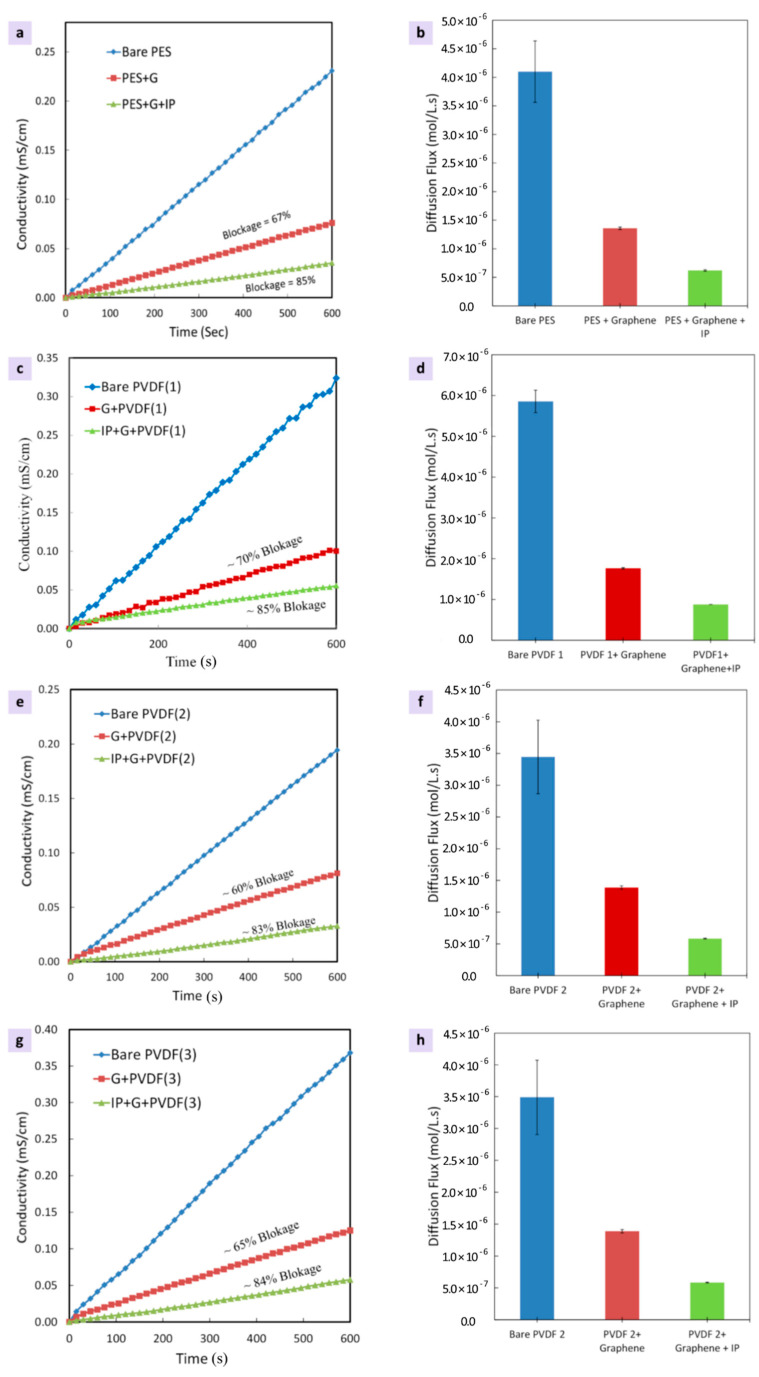
Ionic (KCl) transport studies in terms of conductivity and diffusion flux measurments performed on the developed graphene/hydrophilic composite membranes, (**a**,**b**) for PES, (**c**,**d**) for PVDF 1, (**e**,**f**) for PVDF 3, and (**g**,**h**) for PVDF 3.

In order to measure the quality of the tranferred graphene layer, the trasnport rate of KCl ions was evaluated for all graphene/hydrophilic substrates composites. Figure 10 displays the results of the ions transfer rate experiments. The results show KCl ions rejection of 70% for graphene/PVDF 1, 67% for graphene/PES, 60% for graphene/PVDF 2 and 65% for graphene/PVDF 3 composite membranes. As expected, the roughest substrate (PVDF 2) showed the lowest ions rejection, while the smoothest substrate (PVDF 1) provided the best ions rejection. It is worth mentioning that all hydrophilic substrates utilized in this study were found to provide a better KCl ions rejection when compared to the hydrophobic substrates (polypropylene (PP) and PVDF) utilized in our previously published work [52,53]. It is worth noting that the newly developed electrostatic transfer method offers a viable route for using hydrophilic substrates as it does not involve any mechanical forces that could potentially damage the monolayer graphene layer by inducing additional defects such as tears and cracks.

Nevetheless, the graphene transferred using electrostatic charging method developed in this study was still prone to produce some cracks and tears within graphene monolayer. This was indicated by the transport studies and depicted in the SEM micrographs. Upon sealing these defects using Nylon 6.6 via the IP process, KCl ions rejections of 85%, 85%, 84% and 83% were obtained for graphene/PES, graphene/PVDF 2, graphene/PVDF 1 and graphene/PVDF 3 composite membranes, respectively. Although the rejections were remarkably improved, the composites were still leaking around 15%. It is well-known in literature that 10% leakage arises through intrinsic defects within the graphene monolayer [53]. These intinsic defects, typically induced during the graphene synthesis stage, cannot be sealed via the IP process. The remaining few perecentage (about 5%) of leakage can be sealed by optimizing the Nylon 6.6 IP process parameters. In conclusion, a better control of the CVD process parameters combined with the optimized IP process may lead towards synthesizing an impervious graphene/polymer composite membrane.

## 4. Conclusions

Graphene transfer onto a polymeric substrate, both hydrophobic and hydrophilic, can open the scope towards a wide range of graphene based membrane applications. It is currently challenging to transfer graphene onto hydrophilic substrates using the conventional wet transfer method due to the detachment of the graphene layer from the substrate. In the present study, we confirmed that substrate surface characteristics, mainly surface roughness and wettability, played a major role on graphene transferability. For that, we developed a new protocol to transfer graphene onto hydrophilic polymeric substrates. The developed method utilized electrostatic charging to prevent delamination of graphene from the composite membrane during the wet transfer process. Ionic transport studies, using KCl ions, were performed for testing the developed composite membranes.

The results indicated KCl ions rejections of 70% for graphene/PVDF 1; 67% for graphene/PES, 60% for graphene/PVDF 2% and 65% for graphene/PVDF 3 composite membranes. As expected, the roughest substrate (PVDF 2) showed the lowest ions rejection, while the smoothest substrate (PVDF 1) provided the best ions rejection. It is worth noting that the newly developed electrostatic transfer method offers a viable route for using hydropholic substrates as it does not involve any mechanical forces that could potentially damage the monolayer graphene layer by inducing additional defects such as tears and cracks.

Nevetheless, the graphene transferred using electrostatic charging method developed in this study was still prone to produce some cracks and tears within graphene monolayer. Upon sealing these induced defects using Nylon 6.6 via the IP process, KCl ions rejections of 85%, 85%, 84% and 83% were obtained for graphene/PES, graphene/PVDF 2, graphene/PVDF 1 and graphene/PVDF 3 composite membranes, respectively. Although the rejection was remarkably improved, the composites were still leaking around 15%. Apart from the 10% leakage typically due to the intrinsic defects generated during graphene synthesis, optimizing the IP process parameters could yield 90% rejection. In conclusion, a better control of the CVD process parameters combined with the optimized IP process may lead towards synthesizing an impervious graphene/polymer composite membrane.

## Figures and Tables

**Table 1:** Polymeric substrates characteristics as provided by the supplier.

ID	Substrate	As Received Pore Size (nm)	Thickness (µm)	Wettability
1	PES	20	20	Hydrophilic
2	PVDF 1	10	50	Hydrophilic
3	PVDF 2	10	50	Hydrophilic
4	PVDF 3	10	50	Hydrophilic

**Table 2:** Summary of polymeric substrates’ surface roughness and contact angle.

ID	Substrate	Pore Size (nm) (as Provided by Supplier)	CA (°)	RMS (nm)
1	PES	20	50.0 ± 3.0	5.7 ± 1.2
2	PVDF 1	10	56.5 ± 5.0	4.3 ± 0.5
3	PVDF 2	10	73.5 ± 1.0	32.1 ± 3.6
4	PVDF 3	10	61 ± 2.0	14.5 ± 2.9
